# Cancer‐Associated Adipocytes in Human Breast Cancer: An Observational Histopathological Study of Dedifferentiation and Stromal Transition

**DOI:** 10.1155/tbj/5673713

**Published:** 2026-04-29

**Authors:** Jun Xiao, Xiaomei Zhou, Yichen Wang, Haibin Wu, Hong Zeng, Yiping Wu

**Affiliations:** ^1^ Department of Breast Surgery, Yueyang Central Hospital, 39 Dongmaoling Road, Yueyang, 414000, Hunan, China; ^2^ Department of Plastic and Cosmetic Surgery, Tongji Hospital, Tongji Medical College, Huazhong University of Science and Technology, Wuhan, 430030, China, hust.edu.cn; ^3^ Chinese Medicine Health Management Center, Shenzhen Traditional Chinese Medicine Hospital, Shenzhen, 518033, China, szszyy.cn

**Keywords:** adipocyte, breast cancer, dedifferentiation, metabolism, protumor microenvironment

## Abstract

**Background:**

Adipose tissue is a major stromal component of the breast cancer (BC) tumor microenvironment (TME), playing a crucial role in BC progression. Cancer‐associated adipocytes (CAAs), located at the invasive tumor front, undergo significant morphological and functional alterations. This observational case–control study investigated the dedifferentiation trajectory of CAAs and its impact on BC progression.

**Methods:**

Paired tumor and distant normal adipose tissues from 20 BC patients were analyzed. Histological and immunohistochemical analyses were performed to assess morphological changes and marker expressions α‐SMA, S100A4, and CD36 in CAAs.

**Results:**

CAAs exhibited features consistent with dedifferentiation toward a myofibroblast‐like phenotype, marked by expressing α‐SMA and S100A4, indicators of myofibroblasts and tumor‐associated fibroblasts. Metabolically, CAAs showed increased CD36 expression and histological features compatible with augmented lipolysis and were spatially associated with areas of extracellular matrix (ECM) remodeling. Masson’s trichrome staining demonstrated augmented pericellular collagen deposition, accompanied by increased tissue stiffness and enhanced angiogenesis at the tumor–adipose boundary. In addition, nuclear translocation of β‐catenin in peritumoral adipocytes implicates the Wnt/β‐catenin signaling axis as a potential regulator of adipocyte–mesenchymal transition in this context.

**Significance**
• This observational histopathological study shows that cancer‐associated adipocytes at the invasive front of human breast cancer undergo dedifferentiation with adipocyte–mesenchymal transition–like features. These changes are associated with metabolic reprogramming, stromal remodeling, and a protumor microenvironment, with a possible involvement of Wnt/β‐catenin signaling.

## 1. Introduction

The tumor stroma is a highly heterogeneous tissue containing tumor stem cells and mesenchymal cells, including adipocytes, fibroblasts, and immune cells. The orchestration of cell interactions and metabolic reprogramming in the stroma shapes the protumor microenvironment (TME). Adipose tissue is a major stromal component of the human breast, providing the microenvironment that surrounds the terminal ductal–lobular units, the primary site of mammary carcinogenesis.

Previous studies have defined cancer‐associated adipocytes (CAAs) as adipocytes located at the invasive tumor margin, which is adjacent to tumor cells [1]. Morphologically, CAAs exhibit reduced cell volume and contain smaller, dispersed lipid droplets compared to mature adipocytes [2]. Furthermore, CAAs exhibit an altered secretory profile, releasing adipokines, cytokines, and chemokines that encourage cancer cells to develop a more malignant phenotype. For instance, CAAs isolated from triple‐negative breast cancer (TNBC) patients exhibit upregulated IL‐8 secretion, which has been shown to reshape the immune microenvironment by suppressing CD4+ and CD8+ T‐cell infiltration and upregulating CD274, thereby fostering a protumorigenic state [3]. In addition, CAAs secrete leukemia inhibitory factor, which activates STAT3 signaling in tumor cells and contributes to a feed‐forward loop between CAAs and breast cancer (BC) cells that enhances tumor invasion and metastasis [4].

Beyond secretome alterations, adipocytes at the invasive front exhibit marked phenotypic plasticity under different metabolic conditions. The fate of these reprogrammed adipocytes and their functional impact on tumor progression remain elusive. Bochet et al. reported that adipocytes cocultured with BC cells underwent an elongated fibroblastoid morphology concomitant with preadipocyte factor delta‐like noncanonical Notch ligand 1 (DLK1) induction and depletion of mature adipose markers [5]. These adipocytes can differentiate into fibroblast‐like cells, characterized by enhanced secretion of fibronectin and type I collagen. Furthermore, adipocyte‐derived fibroblasts (ADFs) expressed the cancer‐associated fibroblast (CAF) marker FSP‐1 but did not express α‐SMA. Functionally, ADFs could infiltrate the tumor center and participate in fibroproliferative responses through enhanced migration and invasion capabilities. However, Andreucci et al. mimicked an acidic TME and found that adipose‐derived stem cells exposed to lactic acidosis lost typical adipocyte markers while upregulating myofibroblast markers, such as α‐SMA and COL1A1 [6]. Furthermore, compared to basal acidic‐treated adipose‐derived stem cell conditioned medium (acADSC‐CM), acidic acADSC‐CM significantly enhanced the migratory and invasive capacities of BC cells. These phenotypic and functional alterations indicated that acADSCs transformed CAFs with protumor characteristics.

The above divergent findings revealed that the differentiation direction of CAAs remains unclear, potentially reflecting adipocyte heterogeneity within the TME. Furthermore, existing evidence is predominantly derived from coculture models, and adipocytes cultured with cancer cells in vitro fail to recapitulate the dynamic heterogeneity of the TME in vivo. To date, direct evidence definitively demonstrating adipocyte dedifferentiation within an authentic TME remains elusive. Therefore, this study aimed to comparatively analyze paired BC tissue specimens to define the morphological and molecular characteristics of CAAs relative to mature adipocytes, investigate the fate of CAAs in terms of dedifferentiation and stromal transition within the TME, and evaluate the potential associations between CAA phenotypic alterations and histopathological indicators of tumor progression. This study is intended to provide spatial histopathological evidence for adipocyte phenotypic plasticity in the native BC microenvironment.

## 2. Materials and Methods

### 2.1. Patients and Specimens

Twenty patients with BC were selected from the pathology archives of the Department of Thyroid and Breast Surgery, Yueyang Central Hospital, Hunan Province, between September 2022 and September 2023. Inclusion criteria were as follows: (1) primary BC confirmed by histopathology; (2) treatment‐naïve status (no neoadjuvant therapy) and receipt of curative radical surgery; and (3) availability of matched specimens of tumor and distant normal adipocytes (NAs). Exclusion criteria included the following: (1) concomitant other malignancies; (2) severe dysfunction of major organs; and (3) local or systemic diseases that could affect specimen collection. This study was approved by the Ethics Committee of Yueyang Central Hospital and strictly adhered to the ethical guidelines for biomedical research (Supporting Information 1). This study has obtained the informed consent of the patients. The investigations were conducted in accordance with the Declaration of Helsinki.

Baseline clinical characteristics (Table 1) were retrieved from the Yueyang Central Hospital Health Information System (HIS) using a structured electronic medical record extraction platform. Variables were prespecified according to the study objectives and included age, BMI, tumor histological grade, lymph node status, receptor expression, and Ki67 index. The cutoff for high versus low Ki67 was set at 14%. Continuous variables are summarized as mean ± standard deviation and categorical variables as counts (percentages).

**TABLE 1:** Characteristics of patient samples.

Clinical features	Total cohort (*N* = 20)
Age, year	53.75 ± 11.57
BMI (kg/m²)	
< 18.5	4 (20%)
18.5–24	8 (40%)
> 24	8 (40%)
Tumor size	
≤ 2 cm	9 (45%)
> 2 cm	11 (55%)
Grade	
I‐II	15 (75%)
III	5 (25%)
Lymph node metastasis	
Negative	9 (45%)
Positive	11 (55%)
Estrogen receptor	
Negative	5 (25%)
Positive	15 (75%)
Progesterone receptor	
Negative	6 (70%)
Positive	14 (30%)
HER2	
Negative	6 (70%)
Positive	14 (30%)
Ki67	
Low (< 14%)	7 (35%)
High (≥ 14%)	13 (65%)
Subtypes	
TNBC	4 (20%)
Non‐TNBC	16 (80%)

### 2.2. Hematoxylin and Eosin (H&E) Staining

After fixation in formalin, tissue specimens were embedded in paraffin. Sections were sequentially immersed in xylene I (10 min), xylene II (10 min), absolute ethanol I (5 min), absolute ethanol II (5 min), 95% ethanol (3 min), 90% ethanol (3 min), 80% ethanol (2 min), and 70% ethanol (2 min), followed by rinsing in distilled water for 2 min. Sections were stained in Harris hematoxylin for 5–7 min and blued under running tap water. Differentiation was performed in 1% acid alcohol for 2–5 s, followed by re‐bluing under running water. Sections were then stained in 1% aqueous eosin for 2 min and rinsed in tap water for 30 s. Dehydration was carried out with absolute ethanol, clearing with xylene, and the slides were air‐dried and mounted with neutral resin for microscopic examination.

Images were acquired using a laboratory optical microscope (CX40, SDPTOP Sunny Group Technology) with a 20× objective. For each specimen, five adipocyte fields were selected by systematic random sampling, avoiding overlapping regions and areas with pronounced folds. Photomicrographs were analyzed in ImageJ. The mean adipocyte volume from each image was taken as the value for that field, and the average of the five fields was used to represent the adipocyte volume at the sample level.

### 2.3. Immunofluorescence (IF)

Paraffin sections were baked at 65°C for 3 h and then sequentially immersed in Dewaxing Solution I (15 min), Dewaxing Solution II (15 min), absolute ethanol (5 min), 95% ethanol (5 min, twice), 75% ethanol (5 min), and 50% ethanol (5 min), followed by running water washes (3 min × 3). Sections were immersed in 1× antigen retrieval buffer at 100°C for 30 min, kept warm for 20 min, and then cooled to room temperature together with the retrieval buffer. Sections were washed with PBST three times (5 min each), permeabilized with 0.1% Triton X‐100 for 15 min, and washed with PBST. After blocking with rapid blocking buffer at room temperature for 30 min, primary antibodies were applied and incubated overnight at 4°C. Following three PBST washes, secondary antibodies were applied and incubated at room temperature for 1 h and then washed with PBST. Mounting medium containing DAPI was added, coverslips were applied, and images were acquired under a fluorescence microscope. The following antibodies were used and stained according to the manufacturer’s instructions. The antibodies of perilipin 1 (Cat No. 27716‐1‐AP), α‐SMA (Cat No. 14395‐1‐AP), S100A4 (Cat No. 16105‐1‐AP), CD36 (Cat No. 18836‐1‐AP), β‐catenin (Cat No. 51067‐2‐AP), Ki‐67 (Cat No. 27309‐1‐AP), and CD31 (Cat No. 11265‐1‐AP) were from Proteintech, USA. The antibodies of COL1A1 (Cat No. A1352) were from ABclonal, China.

Tumor cells were identified by nuclear atypia (enlarged, hyperchromatic, and irregular nuclei), increased nuclear‐to‐cytoplasmic ratio, and growth in cohesive nests, cords, or small clusters, sometimes forming gland‐like structures.

Multichannel images were acquired on an upright fluorescence microscope (OLYMPUS, Japan) using 20× or 40× objectives. For each specimen, five fields were selected by systematic random sampling, avoiding overlapping regions and areas with prominent folds or strong autofluorescence. Exposure time, gain, and offset were kept constant across all samples within the same batch for each channel. Images were analyzed in ImageJ to obtain the mean fluorescence intensity for each channel. The mean of the 5 fields from a given specimen was used as the sample‐level IF value for subsequent statistical analyses.

### 2.4. Masson Trichrome Staining

Masson trichrome staining was performed using the Solarbio G1340 kit. Paraffin sections were deparaffinized as described above, stained with Weigert iron hematoxylin for 10 min, differentiated with acidic differentiation solution, and rinsed with water. Sections were blued with Masson bluing solution, rinsed with water, and washed with distilled water for 1 min. Ponceau S–acid fuchsin solution was applied for 5–10 min. During the above steps, a weak acid working solution was prepared at a 2:1 ratio of distilled water to weak acid solution and used for 1‐min washes as indicated. Sections were treated with phosphomolybdic acid solution for 1–2 min, washed with weak acid working solution for 1 min, stained with aniline blue for 1–2 min, and washed with weak acid working solution for 1 min. Rapid dehydration was performed with 95% ethanol, followed by absolute ethanol three times (5–10 s each). Sections were cleared in xylene three times (1–2 min each).

Images were captured with a laboratory optical microscope (CX40, SDPTOP Sunny Group Technology) using a 20× objective. For each specimen, five fields were selected by systematic random sampling, avoiding overlapping regions and areas with pronounced folds. Images were processed in ImageJ to quantify the collagen area fraction. The mean collagen area fraction from each image was taken as the value for that field, and the average across 5 fields per specimen was used as the sample‐level collagen content for analysis.

### 2.5. Statistical Analysis

Statistical analyses were performed using GraphPad Prism 9. For two independent datasets, two‐tailed Student’s *t*‐tests were used. For multiple comparisons, one‐way or two‐way ANOVA with Holm–Sidak post hoc multiple comparisons was applied. Statistical significance was determined based on the number of biological replicates indicated in the figure legends (^∗^ *p* ≤ 0.05, ^∗∗^ *p* ≤ 0.01, ^∗∗∗^ *p* ≤ 0.001).

## 3. Results

### 3.1. Dedifferentiation Phenotype of Adipocytes in BC Infiltration

The cohort consisted of 20 BC tissue samples with a mean age of 53.75 years, and the majority had a BMI within the normal or overweight range (80%). Tumor characteristics showed that more than half of the patients had tumor sizes larger than 2 cm, 75% were estrogen receptor–positive, and the majority were classified as non‐TNBC (80%) (Table 1). It is well‐established that once breaking through the basement membrane, breast tumor cells are directly exposed to the TME, which is predominantly composed of adipocytes. To examine CAA volume alteration, here, we first performed H&E staining on tumor tissues and corresponding distant normal adipose tissues from these 20 BC patients. Representative images displayed that the adipocytes adjacent to the tumor were smaller in size compared to those in distant normal adipose tissue (Figures 1(a), 1(b)). In addition, the morphology of CAAs appeared more elongated compared to normal adipose tissue (Figure 1(a)).

FIGURE 1Dedifferentiation phenotype of adipocytes in BC infiltration. (a) Representative H&E‐stained images of adipose tissue located distantly from the tumor, at the invasive front of tumor, and within the tumor. Black arrows indicate fibroblast‐like CAAs. Scale bar, 100 μm. (b) Mean adipocyte volume of adipocytes located distantly from the tumor, at the invasive front of tumor, and within the tumor. ^∗∗∗^ *p* ≤ 0.001. (c) Mean fluorescence intensities of perilipin 1 and DLK‐1 in CAAs and NAs. ^∗^ *p* ≤ 0.05, ^∗∗^ *p* ≤ 0.01, ^∗∗∗^ *p* ≤ 0.001. (d) Representative immunofluorescence images of the mature adipocyte marker perilipin 1 and the preadipocyte marker DLK‐1 in distant NAs and CAAs at the invasive front of tumor. Scale bar, 100 μm.

**Figure:**
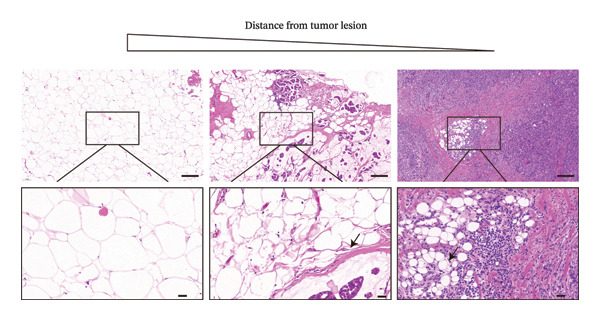
(a)

**Figure:**
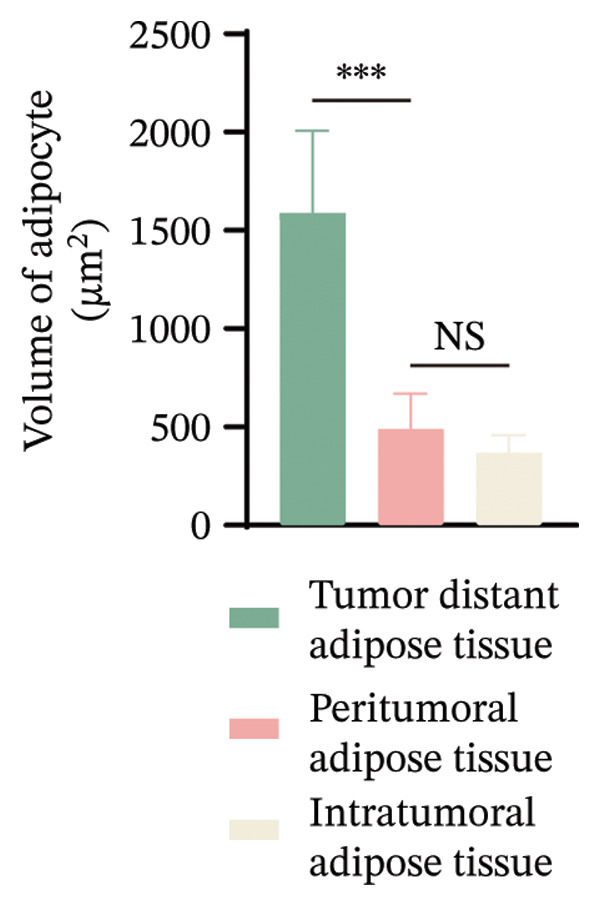
(b)

**Figure:**
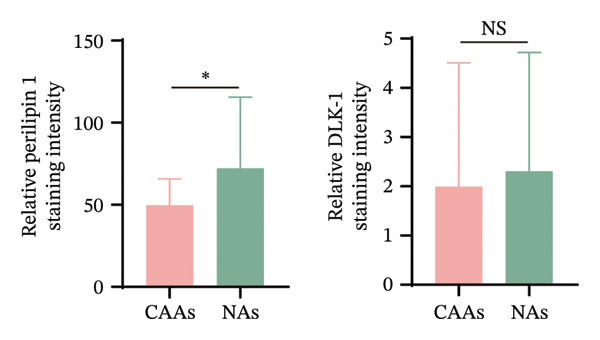
(c)

**Figure:**
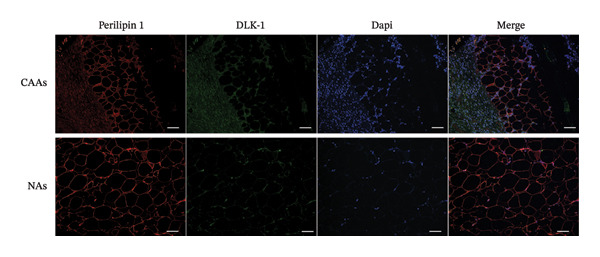
(d)

To further explore the differentiation phenotype of peritumoral adipocytes, we utilized IF to costain perilipin 1 and DLK1 in paired samples (Figure 1(d)). Quantitative statistical analysis revealed that tumor‐adjacent adipocytes exhibited weaker perilipin 1 expression compared to their distant adipose tissue (Figure 1(c)). However, as shown in the representative images, DLK1 expression was not detected in either type of adipose tissue sample. In contrast to these results, Bochet et al. reported time‐dependent upregulation of DLK1 expression in adipocytes upon coculture with BC cells [5]. The observed results may be attributed to differences between in vivo and in vitro experiments or to the limited number of clinical samples. The underlying reasons for this discrepancy warrant further investigation. Besides, the relatively limited sample size precluded meaningful subgroup analyses by tumor subtype. Future explorations with larger, molecularly stratified cohorts will be required to determine whether the adipocyte alterations we observed differ across distinct BC subtypes.

### 3.2. Tumor‐Induced Adipocyte–Mesenchymal Transition (Adi‐MT) in Dedifferentiated Adipocytes

In fact, the expression of mature adipose markers is known to be decreased in adipocytes at the invasive front of tumor, while the expression of preadipocyte markers is not detected. To further investigate the differentiation potential of CAAs, paired clinical specimens were subjected to IF staining for α‐SMA to label myofibroblasts, with perilipin 1 used to specifically identify adipocytes (Figure 2(a)). The results revealed that some peritumoral adipocytes expressed the α‐SMA biomarker, suggesting a transition from adipocytes to myofibroblasts.

FIGURE 2Tumor‐induced adipocyte–mesenchymal transition (Adi‐MT) in dedifferentiated adipocytes. (a) Representative immunofluorescence images of the mature adipocyte marker perilipin 1 and the myofibroblast marker α‐SMA in distant NAs and CAAs at the invasive front of tumor. White arrows indicate CAAs co‐expressing perilipin 1 and α‐SMA, whereas yellow arrows indicate distant NAs located away from the invasive front of tumor that express perilipin 1 only. Scale bar, 100 μm. (b) Representative immunofluorescence images of the mature adipocyte marker perilipin 1 and the cancer‐associated fibroblast marker S100A4 in distant NAs and CAAs at the invasive front of tumor. White arrows indicate CAAs co‐expressing perilipin 1 and S100A4, whereas yellow arrows indicate distant NAs located away from the invasive front of tumor that express perilipin 1 only. Scale bar, 100 μm.

**Figure:**
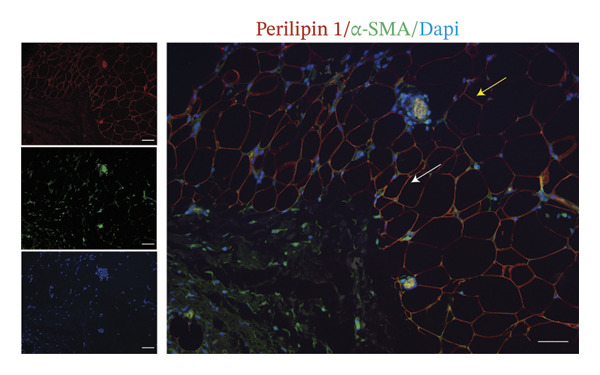
(a)

**Figure:**
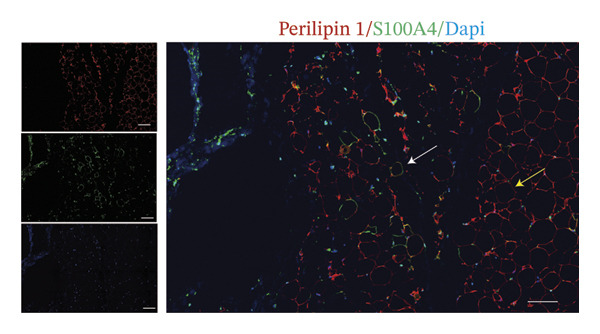
(b)

In addition, some adipocytes showed S100A4 positivity, colocalizing with perilipin 1 in tumor‐surrounding adipose tissue (Figure 2(b)). Although α‐SMA and S100A4 expressions were observed in CAAs at the invasive front, these findings are based on representative images from optimally stained sections, as technical limitations, including variable section quality across this retrospective cohort, precluded systematic quantification for all 20 cases. These results suggested that an alternative route of adipocyte dedifferentiation might be toward CAFs.

### 3.3. CAAs Exhibited Metabolic Alterations

Intriguingly, CAAs are characterized by a reduction in intracellular lipid droplets compared to NAs. At the invasive front of tumors, CAAs facilitate the metabolic demands of BC cells by releasing fatty acids, lactate, and glutamine [7–9]. CD36 is a key membrane protein involved in the transport of fatty acids. To explore the potential mechanisms underlying lipolysis during metabolic reprogramming in CAAs, we performed co‐immunostaining of CD36 and perilipin 1 in paired samples. These results showed that CD36 expression was significantly increased in peritumoral adipose tissue compared to normal adipose tissue (Figures 3(a), 3(b)). Moreover, compared to BC cells within the tumor nests, tumor cells infiltrating into adipose tissue exhibited higher CD36 expression, suggesting a key role for CD36 in fatty acid transport between CAA and BC cells (Figure 3(c)). Tumor cells were identified based on morphological criteria (nuclear atypia, hyperchromasia, and clustering) both in the main tumor nest and at the adipose‐invasive front.

FIGURE 3CAAs exhibited metabolic alterations. (a) Representative immunofluorescence images of the mature adipocyte marker perilipin 1 and CD36 in distant NAs and CAAs at the invasive front of tumor. Scale bar, 200 μm. (b) Mean fluorescence intensity of CD36 in CAAs and NAs. ^∗^ *p* ≤ 0.05, ^∗∗^ *p* ≤ 0.01, ^∗∗∗^ *p* ≤ 0.001. (c) Representative immunofluorescence images of CD36 in carcinoma cells within the tumor and in tumor cells infiltrating adipose tissue. Arrowheads indicate morphologically identified tumor cells based on nuclear atypia and tissue context. Scale bar, 200 μm.

**Figure:**
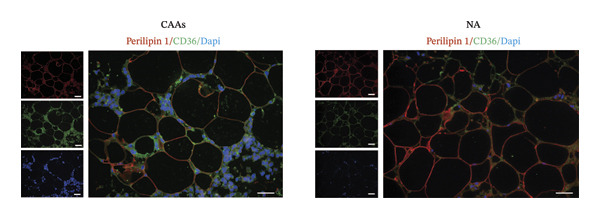
(a)

**Figure:**
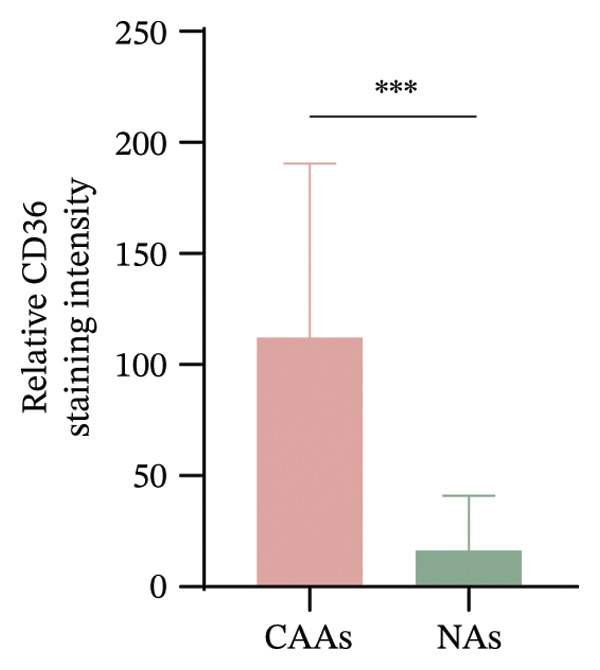
(b)

**Figure:**
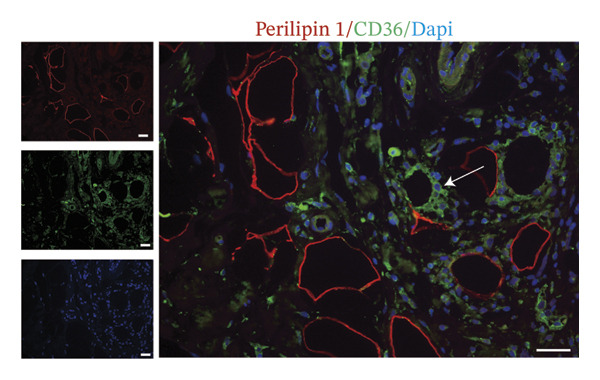
(c)

### 3.4. CAAs Reshaped the Pro‐TME

The extracellular matrix (ECM) constitutes a highly dynamic scaffold that provides both biochemical and biomechanical cues to cancer cells [10]. In BC, adipocyte phenotypic characteristics alter fiber alignment of the ECM, which regulates tumor cell invasion by governing migration persistence [11, 12]. To investigate the role of CAAs in remodeling BC ECM, we performed Masson staining on paired specimens. The results showed that collagen I deposition was detectable even in distant normal adipose tissue (Figure 4(a)). However, quantitative analysis revealed that the mean fibrous area within peritumoral adipose compartments was significantly increased compared to normal controls, suggesting ECM remodeling during tumor invasion (Figures 4(b), 4(c)). Furthermore, focusing on the tumor‐fat interaction area, IF costaining of perilipin and CD31 revealed increased CD31 expression at the invasive front of tumor, suggesting the possibility of high‐density angiogenesis that may create a nutrient‐rich niche to support tumor expansion (Figure 4(d)). At the interface between adipose tissue and tumor tissue, Ki67‐positive tumor cells were frequently observed in close proximity to perilipin 1–positive adipocytes (Figure 4(e)). This spatial position is compatible with potential adipocyte–tumor cell crosstalk, but functional and quantitative studies will be required to determine whether this association has any impact on tumor cell proliferation.

FIGURE 4CAAs reshaped the pro–tumor microenvironment. (a) Representative immunofluorescence images of the mature adipocyte marker perilipin 1 and Col I in distant NAs and CAAs at the invasive front of tumor. Scale bar, 100 μm. (b) Representative Masson trichrome images of distant normal adipose tissue, the invasive front of tumor, and intratumoral adipose tissue. Scale bar, 100 μm. (c) Mean fibrotic area in distant normal adipose tissue and adipose tissue at the invasive front of tumor (*n* = 20). ^∗^ *p* ≤ 0.05, ^∗∗^ *p* ≤ 0.01, ^∗∗∗^ *p* ≤ 0.001. (d) Representative immunofluorescence images of the mature adipocyte marker perilipin 1 and CD31 in distant NAs and CAAs at the invasive front of tumor. Scale bar, 100 μm. (e) Representative immunofluorescence images of the mature adipocyte marker perilipin 1 and Ki‐67 in distant NAs and CAAs at the invasive front of tumor. Scale bar, 100 μm.

**Figure:**
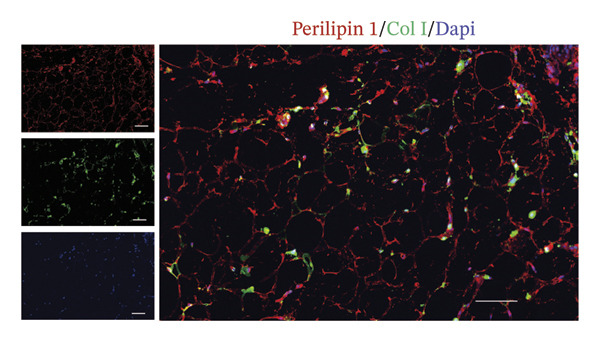
(a)

**Figure:**
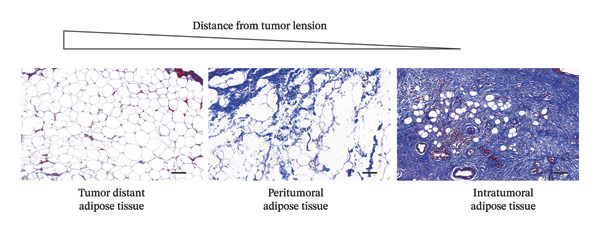
(b)

**Figure:**
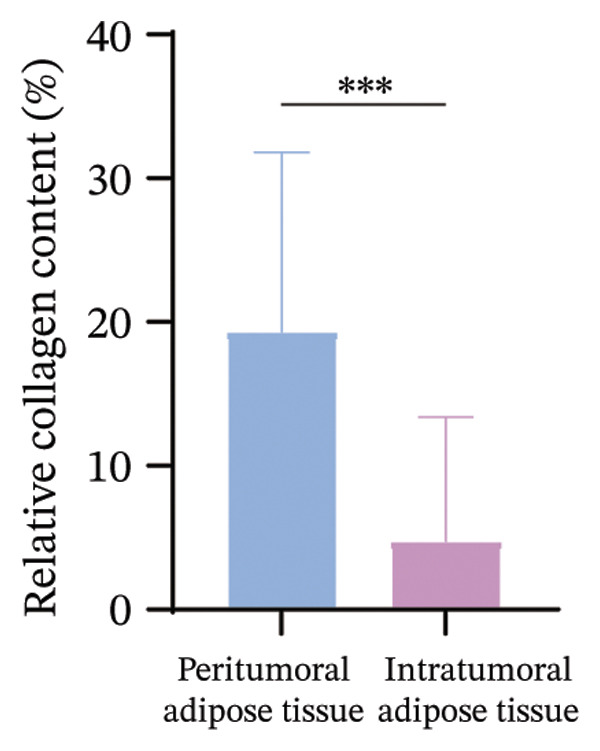
(c)

**Figure:**
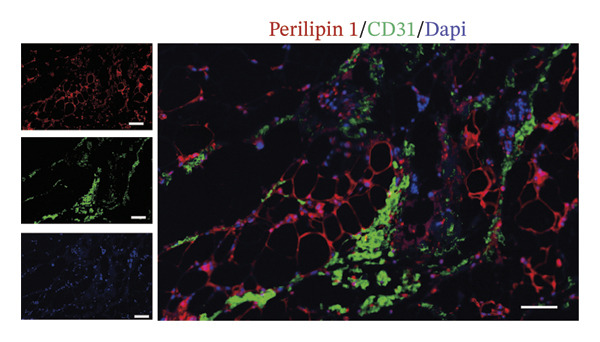
(d)

**Figure:**
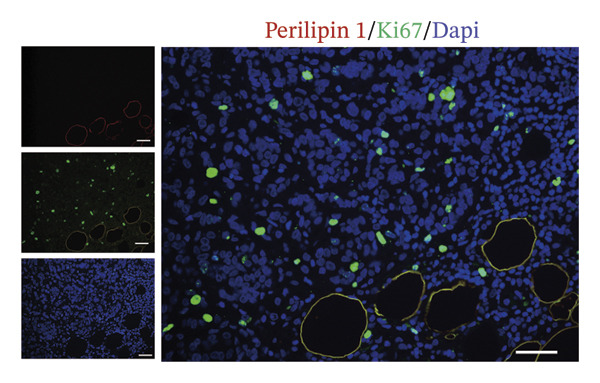
(e)

### 3.5. Wnt/β‐Catenin Signaling Was Associated With Adi‐MT

Among the myriad signaling networks that regulate adipocyte differentiation, the Wnt/β‐catenin pathway plays an important role and is activated in compression‐induced adipocyte dedifferentiation [5, 13]. Because of this, we assessed the activation of the Wnt/β‐catenin pathway in peritumoral adipocytes. The representative images of IF staining revealed that β‐catenin nuclear translocation occurred in some peritumoral adipocytes (Figures 5(a), 5(b)). In contrast, β‐catenin predominantly exhibited membrane localization in adipocytes from distant normal adipose tissue. These results indicated that the Wnt/β‐catenin signaling pathway might be activated and then mediated Adi‐MT at the invasive front of tumor.

FIGURE 5Wnt/β‐catenin signaling was crucial for Adi‐MT. (a) Representative immunofluorescence images of the mature adipocyte marker perilipin 1 and β‐catenin in CAAs at the invasive front of tumor. Scale bar, 40 μm. (b) Representative immunofluorescence images of the mature adipocyte marker perilipin 1 and β‐catenin in distant NAs. Scale bar, 40 μm.

**Figure:**
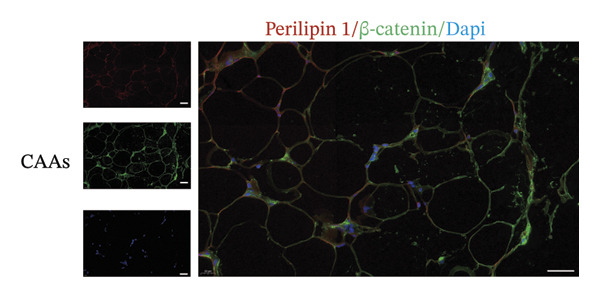
(a)

**Figure:**
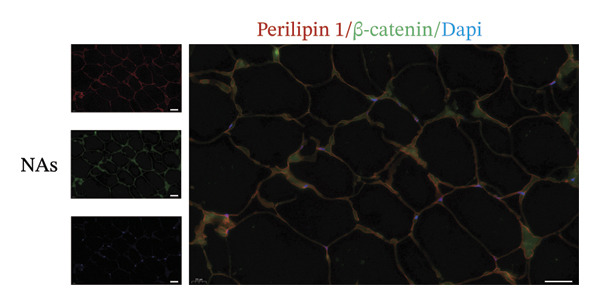
(b)

## 4. Discussion

Under normal conditions, the basement membrane serves as an effective barrier separating the ductal epithelium from the surrounding stromal cells [14]. During breast tumorigenesis, cancer cells breach the basement membrane, enabling direct contact with the adipose‐rich stroma. Adipocytes are not only energy reservoirs but also important endocrine cells [15]. Recent evidence indicates dynamic crosstalk between adipocytes and cancer cells, conferring enhanced migration, invasion, metastasis, and drug resistance capabilities on cancer cells [16]. Within this context, NAs are recruited by tumor cells into CAAs, characterized by reduced volume, decreased lipid droplets, and downregulation of mature adipocyte marker expression [17].

However, there is still controversy regarding the direction of differentiation of CAAs. Adipocytes cocultured with BC cells upregulate the expression of typical preadipocyte markers DLK‐1 [5]. In addition, adipocytes can transition toward a CAF‐like phenotype without expressing α‐SMA. Conversely, single‐cell sequencing analysis demonstrated that dedifferentiated adipocytes could transdifferentiate into α‐SMA‐positive myofibroblasts and macrophage‐like cells, without concomitant upregulation of preadipocyte factor DLK1 [6]. Additionally, our study also emphasized that adipocytes at the invasive front expressed both the myofibroblast marker α‐SMA and the CAF marker S100A4. S100A4 is considered a tumor‐associated fibroblast biomarker involved in tissue remodeling, and S100A4 overexpression has been associated with metastasis and poor prognosis in multiple human malignancies [18]. This indicates that CAAs undergo dedifferentiation and form a heterogeneous, tumor‐promoting cell population, while they do not revert to a typical preadipocyte state.

The interaction between adipocytes and BC cells not only induces phenotypic changes but also profoundly alters the metabolic networks of both cell types. Unlike normal cells, cancer cells preferentially utilize glycolysis over tricarboxylic acid cycle‐mediated oxidative phosphorylation (OXPHOS) for energy production, even under normoxic conditions. This metabolic change is known as the Warburg effect [19]. In addition to glucose, lipids and amino acids are also crucial for highly proliferating cancer cells, including providing the membrane phospholipid components, maintaining membrane fluidity, and serving as sources for lipid signaling and energy production [20, 21]. As the primary source of free fatty acids (FFAs) and triglycerides, adipocytes play a critical role in promoting tumor progression. Accumulating experimental evidence indicates that certain cancer cells can also store excess energy in the form of lipid droplets, which can be further lipolyzed to release FFAs [7, 22, 23]. The transport and intracellular trafficking of FFAs within cancer cells are facilitated by several membrane‐associated binding and transport proteins, including CD36, fatty acid‐binding proteins, and fatty acid transport proteins [24]. In ovarian cancer (OvCa), adipocytes induce CD36 expression in OvCa cells, but not in other types of stromal cells within the omentum. In oral cancer, CD36‐positive cancer cells represent a subpopulation of metastasis‐initiating cells [25]. In our study, we observed heterogeneous CD36 expression levels among BC cells within the primary tumor mass. However, cancer cells infiltrating into adipocyte‐rich regions consistently exhibit markedly stronger CD36 expression. Increasing evidence suggests that the expression of CD36 on the surface of cancer cells is associated with tumor metastasis, chemotherapy resistance, and poor prognosis [26–28]. Adipocyte‐induced CD36 expression in cancer cells has been shown to drive cancer progression, and FA uptake occurs primarily through a CD36‐dependent mechanism.

Furthermore, in our study, compared with NAs, CAAs exhibit stronger CD36 expression. This heightened CD36 expression in lipid‐depleted CAAs presents a seemingly paradoxical observation, as CD36 functions primarily as a unidirectional transporter of FFA. However, this phenomenon could be related to the following mechanisms. In response to the metabolic demands of tumors, CAAs undergo metabolic reprogramming characterized by increased lipolysis. Daquinag et al. cocultured 4T1 cells with either CD36‐WT or CD36‐KO adipocytes and found that 4T1 cells accumulated fewer fatty acids when cocultured with CD36‐KO adipocytes compared to the WT group [29]. Thus, CD36 is essential for fatty acid transport in adipocytes. The inhibition of CD36 function in adipocytes or endothelial cells reduced the mobilization of long‐chain fatty acids and subsequent bioavailability to tumors. Importantly, increased lipolysis triggers CD36 deacylation, resulting in altered subcellular localization to meet the metabolic demands of tumor growth, invasion, and metastasis.

The tumor ECM not only provides cellular attachment but also transmits mechanical and chemical signals, facilitating interactions between different cell types. Collagen, as the principal noncellular component of the ECM, particularly collagen type I, can suppress adipogenic differentiation by inducing YAP nuclear translocation [30]. Notably, the role of the physical properties of the TME in promoting tumor progression has recently attracted widespread attention [13].

The remodeled ECM enhances tumor invasiveness and metastatic potential by exerting physical pressure and altering gene expression and signal transduction. In addition, compression of blood and lymphatic vessels leads to elevated interstitial fluid pressure within the tumor stroma, which in turn impairs the efficacy of immunotherapy, radiotherapy, and chemotherapy [31]. Currently, the detection and quantification of physical abnormalities in the TME provide new strategies for cancer treatment.

Our data demonstrated that the collagen content within adipose tissue at the invasive front of the tumor was higher compared to that distant from the tumor. This augmented collagen deposition is consistent with increased stiffness of the BC ECM, which is a hallmark of desmoplastic remodeling that is closely associated with tumor progression and invasive behavior. Adipocytes remodel tumor ECM stiffness by altering collagen content, and in turn, the altered physical characteristics of the TME also modulate the biological behavior of adipocytes. Under in vitro hyperosmotic conditions mimicking aspects of the TME, compression‐induced dedifferentiated adipocytes (CiDAs) dedifferentiate into pluripotent cells, which are capable of osteogenic, myogenic, and chondrogenic differentiation when cultured in respective induction media. These CiDAs can further differentiate into protumorigenic myofibroblasts, thereby promoting BC progression [13].

Adipocytes cocultured with BC cells exhibited elevated β‐catenin expression and acquired an FSP‐1^+^ α‐SMA^-^ CAF phenotype [5]. Our results demonstrate increased nuclear translocation of β‐catenin at the tumor–adipose interface, suggesting that the Wnt/β‐catenin signaling pathway regulates adipocyte dedifferentiation in this context. The Wnt/β‐catenin signaling pathway, known as an inhibitory regulator of adipogenesis, has been shown to play an important role in the dedifferentiation and transdifferentiation of adipocytes. In OvCa, cancer cells promote adipocyte dedifferentiation by activating the Wnt/β‐catenin signaling pathway, and the omental ADFs exhibit protumor characteristics [32].

Lastly, we should admit some issues in terms of our study. Firstly, this study is based on a relatively small cohort of 20 BC patients, which may not capture the full heterogeneity of CAAs across different patient populations. A larger sample size could provide more robust and generalizable findings. Secondly, the study identifies phenotypic and molecular changes in CAAs and does not include functional assays to directly demonstrate the impact of these changes on BC progression. The study primarily uses paraffin‐embedded human tissue samples, lacking in vivo experimental validation. Animal models could be employed to verify the findings and explore the mechanistic pathways in a controlled setting. Further ongoing explorations could strengthen the causal links between CAAs and BC tumor behavior.

## 5. Conclusion

In conclusion, this study systematically highlights the Adi‐MT observed in BC samples during the interactions between BC cells and CAAs. CAAs are associated with metabolic reprogramming and ECM collagen deposition, features that may reshape the TME and favor angiogenesis and BC cell proliferation. Further functional studies are required to establish causal relationships. Our findings suggest potential new directions for targeted therapeutic strategies. However, our study is limited to analyses based solely on paraffin‐embedded human BC tissue samples, relying primarily on molecular marker detection at the tissue level, without experimental validation at cellular or animal levels. More explorations are necessary to integrate multilevel approaches to further decipher the precise mechanisms underlying the interactions between BC cells and adipocytes.

**Nomenclature**
acADSC‐CM: Acidic‐treated adipose‐derived stem cell conditioned medium
ADFs: Adipocyte‐derived fibroblasts
Adi‐MT: Adipocyte–mesenchymal transition
BC: Breast cancer
CAAs: Cancer‐associated adipocytes
CAFs: Cancer‐associated fibroblasts
CiDAs: Compression‐induced dedifferentiated adipocytes
DLK1: Delta‐like noncanonical Notch ligand 1
ECM: Extracellular matrix
FFAs: Free fatty acids
H&E: Hematoxylin and eosin
IF: Immunofluorescence
NAs: Normal adipocytes
OvCa: Ovarian cancer
OXPHOS: Oxidative phosphorylation
TME: Tumor microenvironment
TNBC: Triple‐negative breast cancer

## Author Contributions

Conceptualization, Hong Zeng; writing–original draft preparation, Xiaomei Zhou; writing–review and editing, Jun Xiao and Xiaomei Zhou; supervision, Yiping Wu; project administration, Haibin Wu; and investigation, Yichen Wang.

## Funding

This work was supported by the Hunan Provincial Natural Science Foundation of China (2023JJ50305), Wu Jieping Medical Foundation (320.6750.2023‐18‐88), and China Postdoctoral Science Foundation (2023M741278).

## Conflicts of Interest

The authors declare no conflicts of interest.

## Supporting Information

Ethical approval documents can be found in Supporting Information 1.

## Supporting information


**Supporting Information** Additional supporting information can be found online in the Supporting Information section.

## Data Availability

The corresponding author could be contacted for data inquiries. All data generated or analyzed during this study have been included in this article.
