# Supplementary material for: Cancer‐Associated Adipocytes in Human Breast Cancer: An Observational Histopathological Study of Dedifferentiation and Stromal Transition
Source: Breast J. 2026 Apr 29;2026:5673713. doi: 10.1155/tbj/5673713 (PMC13126615; doi:10.1155/tbj/5673713)
Supplement: Supplementary file 1 — Supporting Information Additional supporting information can be found online in the Supporting Information section. [file TBJ-2026-5673713-s001.pdf]

岳阳市中心医院医学伦理委员会

伦理审查批件

审查批件号	岳市中心医院医伦审第（2022-045）号		
研究项目名称	乳腺癌中与癌症相关的脂肪细胞去分化现象及相关机制研究		
研究分类	☐干预性研究 ☑观察性研究 ☐其他________		
项目来源	院内科研项目		
性质	☐多中心（☐国际 ☐国内）<br>☑单中心	是否需遗传办审批	否
项目负责人	肖君	实施科室	乳腺科
审查类别	初始审查	审查方式	简易审查
审查日期	2022 年 11 月 04 日	审查地点	院本部行政楼三楼会议室
审查文件	☑研究方案（1.0/2022 年 10 月 10 日）<br>☑知情同意书（1.0/2022 年 10 月 10 日）<br>☑其他 初始审查申请、项目负责人简历、科研诚信承诺书、项目负责人经济利益声明、知识产权归属说明、资料真实性、一致性承诺书、研究项目经费来源说明、知识产权归属说明、人类遗传资源管理承诺书		

审查结论

根据原国家卫生和计划生育委员会《涉及人的生物医学研究伦理审查办法(2016 年)》、NMPA《药物临床试验质量管理规范(2020 年)》、《药物临床试验伦理审查工作指导原则(2010 年)》、《医疗器械临床试验质量管理规范(2016 年)》、WMA《赫尔辛基宣言(2013 年)》和 CIOMS《人体生物医学研究国际道德指南》的伦理原则，经本伦理委员会审查，**批准**开展本项研究。

年度/定期跟踪审查频率	12 个月
批件有效期	12 个月
主任委员/副主任委员签字	夏[illegible]
签发日期	2022.11.8
伦理委员会	岳阳市中心医院医学伦理委员会<br>（盖章）<br>岳阳市中心医院 医学伦理委员会 43060210020775

地址/邮编	湖南省岳阳市东茅岭路39号、岳阳大道28号<br>邮编：414000
联系电话	0730-8246502　0730-8750562
附　件	简易审查综合意见

声　明

本伦理委员会的职责、人员组成、操作程序及记录遵循GCP、符合赫尔辛基宣言的原则、并遵守中国相关法律和法规的规定。

注意事项：

1、研究应遵循GCP原则和国家法律法规的要求、按照本伦理委员会批准的方案开展研究，保护受试者的健康与权益。

2、凡涉及采集、保藏、利用、对外提供我国人类遗传资源或者按照国家规定必须经有关部门专项审批的内容，均需在项目执行前向有关部门申报并获得批准，并及时递交相关文件备案。

3、研究过程中，若变更项目负责人，对研究方案、知情同意书等的任何修改，请提交修正案审查申请，获得伦理委员会批准后方可执行。

4、本伦理委员会对研究项目进行定期跟踪审查，自批件签发之日起，请研究者在规定的定期跟踪审查截止日期前1个月提交研究进展报告；在批件有效期内未启动研究项目，请及时提交研究进展报告，申请延长批件有效期，如未及时申请，逾期批件自行废止。

5、本研究中心发生的SAE，研究者应在获知后24小时内以严重不良事件报告表的形式报告伦理委员会。其他应及时报告的安全性报告包括可疑且非预期严重不良反应报告、其他潜在的严重安全性风险信息的报告、年度安全性报告等。

6、研究者必须及时报告研究实施中为消除对受试者紧急危害的研究方案的偏离或者修改、增加受试者风险或者显著影响研究实施的改变；其他违背方案应在发现后1个月内及时提交违背方案报告。

7、暂停或提前终止研究应及时提交暂停/终止研究报告、暂停或提前终止研究所取得的结果的总结（如有）。

8、完成研究，请提交本中心结题报告、研究总结报告（适用多中心研究）。

9、及时报告其他伦理委员会的重要决定（适用多中心研究）。

10、按审查意见修改后的文件，或对审查意见不同观点的陈诉，请提交“复审申请”，方案/知情同意书需注明新的版本号和版本日期，并以前后对照的形式注明修改明细，报伦理委员会审查，经批准后执行。

附件　YYCH-YXLL-SOP-FJ-017·01

岳阳市中心医院医学伦理委员会

简易审查综合意见

伦理审查受理号	2022-045		
研究项目名称	乳腺癌中与癌症相关的脂肪细胞去分化现象及相关机制研究		
方案版本号	1.0	版本日期	2022 年 10 月 10 日
知情同意书版本号	1.0	版本日期	2022 年 10 月 10 日
初审日期	2022 年 11 月 04 日	上一次审查日期	NA
申请专业	乳腺科	主要负责人	肖君
主审委员	夏伟瑜、郭忠	审查类别	初始审查
主审意见：<br>☑ 批准　☐ 修改后批准　☐ 修改后再审　☐ 不批准　☐ 暂停或终止研究			
☐ 转会议审查			
调整跟踪审查频率	☐ 不变　☐ 改变<br>☑ 不适用	修正跟踪审查频率	______个月
审查流程的安排	☑ 提交会议报告　☐ 提交会议审查		
伦理委员会秘书签名	[signature]		
日期	2022.11.08		

岳阳市中心医院 医学伦理委员会 430602100020775
